# Congenital Bile Acid Synthesis Defect Type 2 Mimicking Biliary Atresia: A Case Report of Delta-4-3-Oxosteroid 5-Beta-Reductase Deficiency in a Two-Month-Old Infant

**DOI:** 10.7759/cureus.116257

**Published:** 2026-09-15

**Authors:** Mariam Lagrine, Douaa Kaouani, Rabiy Elqadiry, Houda Nassih, Aicha Abourrahouat, Imane Aitsab

**Affiliations:** 1 Department of Pediatrics B, Mother and Child Hospital, Mohammed VI University Hospital, Marrakech, MAR; 2 Department of Pediatrics, Mohammed VI University Hospital, Marrakech, MAR; 3 Department of Pediatrics, Mother and Child Hospital, Mohammed VI University Hospital, Faculty of Medicine and Pharmacy, Cadi Ayyad University, Marrakech, MAR; 4 Department of Pediatrics, Mother and Child Hospital, Mohammed VI University Hospital, Marrakech, MAR

**Keywords:** akr1d1, bile acid synthesis defect, biliary atresia, inborn error of metabolism, neonatal cholestasis, δ4-3-oxosteroid 5β-reductase deficiency

## Abstract

Neonatal cholestasis presents a complex diagnostic challenge, frequently necessitating the differentiation of numerous etiologies, including the anatomically correctable biliary atresia (BA), from various metabolic and genetic disorders. The timely and accurate identification of inborn errors of bile acid metabolism is particularly critical, as these conditions can lead to progressive liver damage if left untreated.

Congenital bile acid synthesis defects (CBASDs) are rare autosomal recessive inborn errors of metabolism that can manifest as neonatal cholestasis. Their clinical and biochemical profile, particularly in cases of normal or low gamma-glutamyl transferase (GGT) in the setting of significant cholestasis, can mimic other causes of neonatal liver disease, most notably BA, thereby posing a significant diagnostic challenge.

We report a two-month-old male infant presenting with early-onset cholestatic jaundice since day 20 of life. Clinical and ultrasonographic findings initially raised strong suspicion for BA. However, the biochemical profile of markedly elevated transaminases, cholestasis with normal-to-low GGT, massive hyperferritinemia, and relatively low total bile acids suggested a metabolic etiology.

Urine bile acid profiling by liquid chromatography-tandem mass spectrometry revealed trace levels of primary bile acids with accumulation of Δ4-3-oxo intermediates. Next-generation sequencing has confirmed the diagnosis of CBASD type 2. Liver biopsy demonstrated giant cell hepatitis without ductal proliferation, thus definitively excluding BA and precluding the need for a Kasai portoenterostomy.

## Introduction

Congenital bile acid synthesis defects (CBASDs) are rare autosomal recessive disorders caused by enzymatic defects in the primary bile acid biosynthetic pathway, representing a potentially treatable yet diagnostically challenging cause of progressive neonatal cholestasis and liver failure [1,2]. CBASD type 2, caused by AKR1D1-encoded Δ4-3-oxosteroid 5β-reductase deficiency, disrupts a critical step in steroid ring structure modification during bile acid synthesis [3]. A hallmark biochemical feature is normal or reduced GGT despite conjugated hyperbilirubinemia, a key discriminator from biliary atresia (BA), whose misrecognition may lead to unnecessary surgical intervention and delayed life-saving treatment [4]. We report a two-month-old male infant with CBASD type 2 whose initial presentation closely mimicked BA, underscoring the indispensable role of metabolic and genetic testing in the workup of neonatal cholestasis.

## Case presentation

A two-month-old male infant, born at term following an uneventful pregnancy, presented for the evaluation of persistent neonatal cholestatic jaundice. Jaundice was first noted on the 20th day of life, accompanied by partially acholic stools and dark urine. The parents were second-degree relatives, which was highly relevant given the suspected autosomal recessive inheritance. There was no family history of neonatal cholestasis, unexplained infant death, or liver disease in the extended family.

Upon admission, the infant appeared jaundiced but in good general condition, exhibiting age-appropriate weight gain. Vital signs were stable (heart rate: 140 bpm, respiratory rate: 35 breaths/min, blood pressure: 85/50 mmHg). Growth parameters were at the 50th percentile (weight: 3.9 kg, length: 52 cm). Physical examination revealed a firm liver edge palpable 3 cm below the right costal margin. Splenomegaly, ascites, and peripheral edema were absent; the neurological examination and developmental milestones were appropriate for age. 

Abdominal ultrasonography revealed a small, suboptimally filled gallbladder with incomplete postprandial emptying (Figure 1). These findings, alongside early-onset cholestatic jaundice, initially raised strong suspicion for BA. However, ultrasonographic gallbladder abnormalities can occur secondary to reduced bile flow in severe metabolic hepatopathies and do not inherently indicate extrahepatic biliary obstruction.

**Figure 1:**
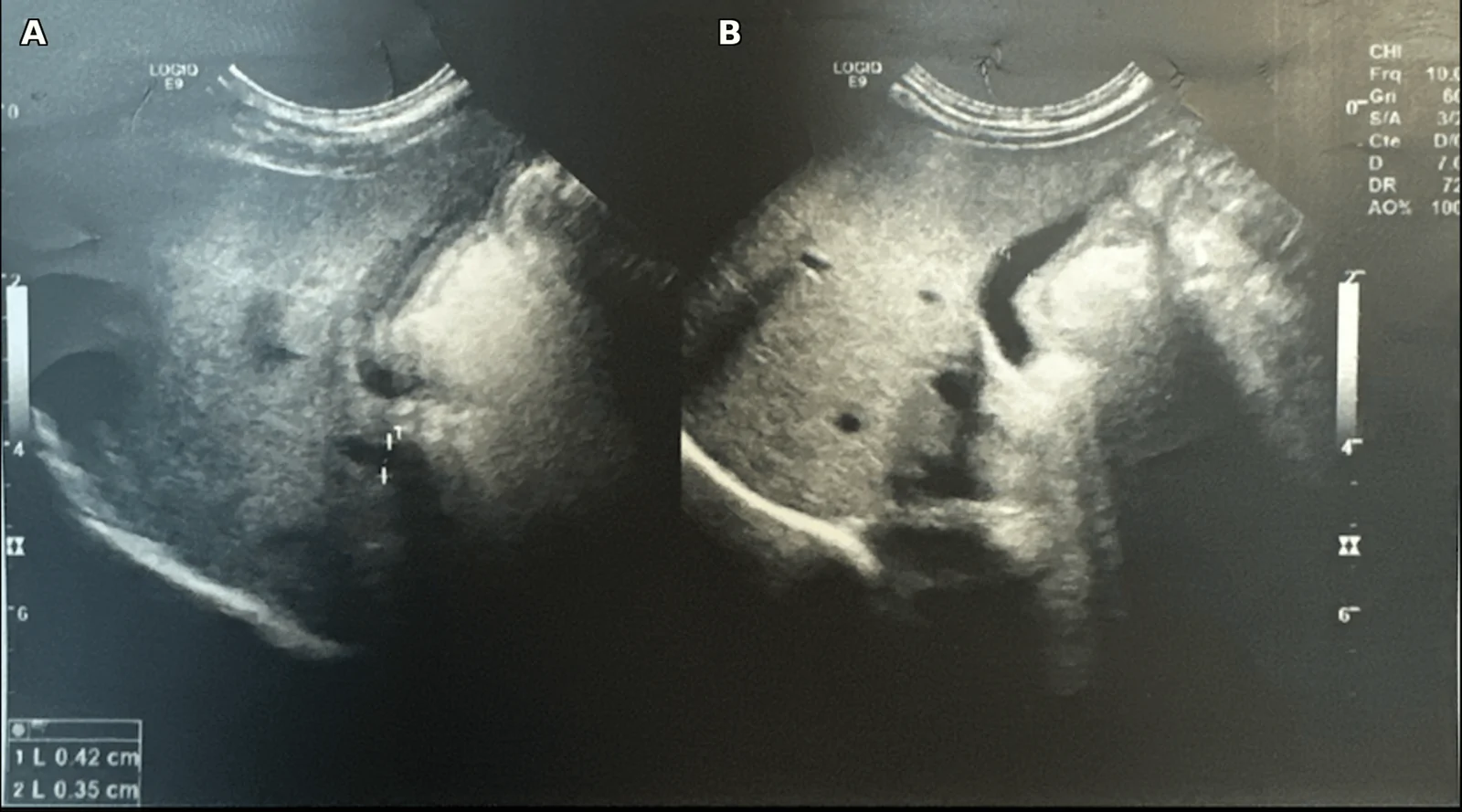
Initial abdominal ultrasonography (A) Longitudinal view of the portal vein demonstrating normal caliber, without periportal thickening or a triangular cord sign. (B) View of the gallbladder showing suboptimal filling and lack of postprandial contraction, features that can mimic biliary atresia but occur secondary to impaired bile flow.

Laboratory investigations revealed a biochemical profile inconsistent with BA and highly suggestive of a metabolic hepatopathy. Marked hepatocellular injury was present, with alanine aminotransferase (ALT) at 1242 U/L and aspartate aminotransferase (AST) at 1751 U/L (over 30 to 40 times the upper limit of normal). Cholestasis was confirmed with a total bilirubin of 254 µmol/L and a direct fraction of 204 mmol/L. Notably, the gamma-glutamyl transferase (GGT) level remained low throughout hospitalization (62-86 U/L), serving as a crucial discriminator against BA.

Serum total bile acids were only mildly elevated (8 to 42 umol/L on day 58), which is disproportionately low relative to the degree of conjugated hyperbilirubinemia and reflects a primary synthetic failure rather than biliary excretion impairment. Impaired hepatic synthetic function was evidenced by a prothrombin time (PT) of 57.6- 58%. Marked hyperferritinemia was noted, peaking at 10 082 ng/ml before decreasing spontaneously to 7514 ng/ml without specific iron chelation. This peak was interpreted as a transient marker of acute, massive hepatocellular necrosis rather than primary iron overload. Alkaline phosphatase (ALP) was at 1062 U/L, while marked elevations can reflect acute hepatocellular injury; this level was interpreted as a combination of hepatic release and high physiological bone turnover characteristic of early infancy. The evolution of laboratory findings is summarized in Table 1.

**Table 1:** Laboratory results of the patient at admission and during follow-up AST: aspartate aminotransferase; ALT: alanine aminotransferase; GGT: gamma-glutamyl transferase

Parameter (Units)	Day 49 of Life	Day 52 of Life	Day 54 of Life	Day 58 of Life	Reference Range
Total bilirubin (µmol/L)	254.91	172.71	—	—	< 17.1
Direct (conjugated) bilirubin (µmol/L)	204.49	123.95	—	—	< 5.1
AST/GOT (U/L)	1751	1170	—	—	5–34
ALT/GPT (U/L)	1242	988	—	—	< 55
GGT (U/L)	62	—	86	78	12-120
Alkaline phosphatase (U/L)	1062	872	—	—	150–500
Prothrombin time (%)	58	57.6	72	89	70–100
Ferritin (ng/mL)	10 082	—	—	7 514	20–200
Total bile acids (µmol/L)	—	—	8	8	1.5–10

The definitive diagnosis was established through urine bile acid metabolite profiling via liquid chromatography-tandem mass spectrometry (LC-MS/MS) and molecular testing. LC-MS/MS identified an accumulation of toxic Delta-4-3-oxo-R bile acid intermediates alongside marked depletion of primary bile acids. Whole exome sequencing identified a homozygous nonsense variant in AKR1D1 (c.286G>T, p.Glu96Ter), confirming CBASD type 2 (Table 2).

**Table 2:** Biochemical and molecular confirmation of bile acid synthesis defect CBASD: Congenital bile acid synthesis defect

Parameter	Result	Reference range	Interpretation
Total serum bile acids (TBA)	7 μmol/L	1.5–10 μmol/L	Disproportionately low relative to degree of cholestasis
Urinary primary/secondary/tertiary bile acids (total)	4.20 μmol/L	Predominant urinary bile acid fraction (normally >90–95% of total profile)	Marked deficiency of normal bile acid conjugates
Urinary Δ4-3-oxo-R bile acid intermediates	Increased concentration	Not normally detected	
*AKR1D1* gene sequencing (Whole Exome Sequencing)	Homozygous pathogenic variant: c.286G>T (p.Glu96Ter), NM_005989.4 / NP_005980.1		Nonsense variant (stop-gained); confirms diagnosis of CBASD type 2

## Discussion

CBASD type 2, caused by pathogenic variants in AKR1D1, is an exceptionally rare autosomal recessive disorder of primary bile acid synthesis. The enzyme Δ4-3-oxosteroid 5β-reductase catalyzes the reduction of the Δ4-3-oxo group on the steroid nucleus, a key step in the conversion of cholesterol to the primary bile acids cholic acid and chenodeoxycholic acid [5]. Enzyme deficiency leads to the accumulation of Δ4-3-oxo bile acid intermediates, which are directly hepatotoxic, and to a concomitant deficiency of normal bile acid conjugates required for bile flow [6].

However, the clinical overlap between bile acid synthesis defects and other cholestatic conditions, particularly BA, often leads to diagnostic delays and inappropriate interventions [7]. This diagnostic ambiguity underscores the need for advanced investigative modalities capable of precisely characterizing the underlying pathophysiology, especially considering that certain genetic defects, such as homozygous mutations in oxysterol 7α-hydroxylase, can lead to rapid progression to cirrhosis [8].

The key biochemical discriminator is the paradox of cholestasis with normal or low GGT. In most cholestatic conditions, elevated GGT reflects increased GGT expression driven by the accumulation of normal bile acids within the biliary tree. In CBASDs, because primary bile acids are lacking, GGT induction does not occur, yielding normal or low serum GGT levels despite obvious jaundice [9].

Ultrasonographic findings, such as a small or non-contracting gallbladder, are frequent in BA but are not entirely specific. In this infant, decreased bile output due to a synthetic block caused secondary gallbladder collapse. Recognizing that low GGT contradicts classic BA helps prevent unnecessary explorative laparotomy or Kasai procedures. 

The profound hyperferritinemia observed (peaking at 10,082 ng/mL) represents another diagnostic pitfall. While extreme ferritin levels in neonates can raise concern for neonatal hemochromatosis or hemophagocytic lymphohistiocytosis, the spontaneous drop in ferritin accompanying transaminase improvement indicates that the hyperferritinemia was driven by acute, extensive hepatocellular cytolysis. Severe necrosis releases intracellular ferritin stores into circulation; as acute inflammation subsides, ferritin levels fall without requiring targeted iron clearance [7,8].

Definitive diagnosis requires urine bile acid analysis by LC-MS/MS followed by molecular confirmation. Identifying the homozygous AKR1D1 variant (p.Glu96Ter) confirmed the diagnosis and facilitated family counseling. Early diagnosis is vital because CBASD type 2 responds remarkably well to primary bile acid replacement therapy (e.g., cholic acid). Primary bile acids restore bile flow and downregulate CYP7A1 via negative feedback, thereby reducing the production of toxic intermediate metabolites and halting progression to end-stage liver disease.

From a therapeutic standpoint, CBASD type 2 is one of the few inborn errors of metabolism amenable to specific oral replacement therapy with primary bile acids. Supplementation suppresses the endogenous production of toxic intermediates via feedback inhibition of cholesterol 7α-hydroxylase (CYP7A1), while simultaneously restoring bile flow. Early diagnosis and treatment initiation are critical determinants of outcome, with liver transplantation potentially avoidable if treatment is commenced prior to the onset of end-stage liver disease [6,9].

## Conclusions

We report a two-month-old infant with CBASD type 2 (Δ4-3-oxosteroid 5β-reductase deficiency) caused by a homozygous nonsense variant in AKR1D1 (p.Glu96Ter), presenting with neonatal cholestasis that clinically and sonographically mimicked BA. The combination of low-GGT cholestasis, marked transaminase elevation, and disproportionately low serum bile acids directed the workup toward a metabolic etiology, avoiding inappropriate surgery. Integration of GGT-based biochemical stratification, urinary LC-MS/MS bile acid profiling, and targeted genetic testing is recommended for all neonates presenting with unexplained low-GGT cholestasis.
